# A novel adaptation of a parent–child observational assessment tool for appraisals and coping in children exposed to acute trauma

**DOI:** 10.3402/ejpt.v7.31879

**Published:** 2016-09-20

**Authors:** Meghan L. Marsac, Nancy Kassam-Adams

**Affiliations:** 1 The Center for Injury Research and Prevention, The Children's Hospital of Philadelphia, Philadelphia, PA, USA; 2 Department of Psychiatry, University of Pennsylvania, Philadelphia, PA, USA; 3 Department of Pediatrics, Kentucky Children's Hospital, Lexington, KY, USA; 4 College of Medicine, University of Kentucky, Lexington, KY, USA; 5 Department of Pediatrics, Perelman School of Medicine, University of Pennsylvania, Philadelphia, PA, USA

**Keywords:** Parent–child interaction, trauma, PTSD, coping, appraisals, assessment, recovery

## Abstract

**Background:**

Millions of children worldwide are exposed to acute potentially traumatic events (PTEs) annually. Many children and their families experience significant emotional distress and/or functional impairment following PTEs. While current research has begun to highlight a role for early appraisals and coping in promoting or preventing full recovery from PTEs, the exact nature of the relationships among appraisals, coping, and traumatic stress reactions as well as how appraisals and coping behaviors are influenced by the child's environment (e.g., parents) remains unclear; assessment tools that reach beyond self-report are needed to improve this understanding.

**Objective:**

The objective of the current study is to describe the newly created Trauma Ambiguous Situations Tool (TAST; i.e., an observational child–parent interview and discussion task that allows assessment of appraisals, coping, and parent–child processes) and to report on initial feasibility and validation of TAST implemented with child–parent dyads in which children were exposed to a PTE.

**Method:**

As part of a larger study on the role of biopsychosocial factors in posttraumatic stress reactions, children (aged 8–13) and parents (*n*=25 child–parent dyads) completed the TAST during the child's hospitalization for injury.

**Results:**

Children and parents engaged well with the TAST. The time to administer the TAST was feasible, even in a peri-trauma context. The TAST solicited a wide array of appraisals (threat and neutral) and coping solutions (proactive and avoidant). Forced-choice and open-ended appraisal assessments provided unique information. The parent–child discussion portion of the TAST allowed for direct observation of parent–child processes and demonstrated parental influence on children's appraisals and coping solutions.

**Conclusions:**

The TAST is a promising new research tool, which may help to explicate how parents influence their child's developing appraisals and coping solutions following a PTE. More research should examine the relationships of appraisals, coping, and parent–child processes assessed by the TAST with traumatic stress outcomes.

**Highlights of the article:**

- The Trauma Ambiguous Situations Tool (TAST) is a promising method to assess children’s appraisals and coping solutions and how parents influence these following a potentially traumatic event.
- The use of open-ended and forced-choice response sets yielded different results regarding child’s appraisals of events as either neutral or threatening.
- More research is needed to determine how the TAST assessment aligns with child health outcomes (e.g., current or subsequent traumatic stress reactions).

Every year, millions of children are exposed to potentially traumatic events (PTEs). In a recent systematic review, Price, Kassam-Adams, Alderfer, Christofferson, and Kazak (2016) identified that approximately 30% of youth and their parents develop significant posttraumatic stress symptoms (PTSS) following exposure of a PTE related to medical events. Following exposure to trauma (i.e., across types such as medical, disaster, child maltreatment, war exposure, and domestic violence), about 16% of children develop full posttraumatic stress disorder (PTSD; Alisic et al., 2014). Theoretical models and empirical investigations have identified early cognitive appraisals and coping behaviors as potential mechanisms of action in the development of PTSS in children (Dalgleish, Meiser-Stedman, & Smith, 2005; Ehlers & Clark, 2000; Marsac, Kassam-Adams, Delahanty, Widaman, & Barakat, 2014; Marsac et al., 2016; Meiser-Stedman, 2002). While some studies have suggested that parent reactions (such as PTSS and depressive symptoms) following PTEs may affect child reactions, the process through which this may occur remains unclear (Alisic, Jongmans, Van Wesel, & Kleber, 2011; Trickey, Siddaway, Meiser-Stedman, Serpell, & Field, 2012). To date, research on appraisals and coping in children exposed to PTEs has primarily utilized self-report methodology. Further, few studies have systematically observed the process of interaction between children and parents immediately following a PTE (Gewirtz, Forgatch, & Weiling, 2008).

Information-processing models of anxiety and traumatic stress highlight the roles of appraisals and coping in the development and persistence of symptoms. Appraising a PTE as threatening can lead to behavioral strategies (i.e., coping solutions such as avoidance) that directly contribute to PTSS and/or prevent the development of longer-term realistic and adaptive appraisals (Ehlers & Clark, 2000; Meiser-Stedman, 2002). Marsac et al. (2014) proposed a biopsychosocial theoretical model focusing on the role of peri-trauma processes during acute medical events. This model specifies a role for biological, psychological, and social factors as having both independent and interactional relationships that influence the development and maintenance of PTSS. In addition to highlighting a role for child appraisals and coping, Marsac et al. (2014) suggest that parent–child interactions during the peri-trauma period may influence children's development of appraisals and coping related to the PTE, in turn influencing long-term PTSS.

A growing evidence base offers support for these models. In examining specific types of appraisals in youth with injuries, perception of threat, negative appraisals about vulnerability to future harm, and negative interpretation of intrusive memories and rumination have been found to be related to worse PTSS (Bryant, Salmon, Sinclair, & Davidson, 2007; Stallard & Smith, 2007). Hitchcock Ellis, Williamson, and Nixon (2015) expanded on the role of appraisals in predicting PTSS, finding that appraisals mediated the relationship between social support and PTSS in youth who had experienced a single-incident PTE. Though the exact nature of the relationship remains unclear, coping has likewise emerged as a potential contributor to PTSS in children. For example, following a motor-vehicle crash, children with PTSD used more coping strategies overall (particularly avoidant/escape strategies) than children without PTSD (Stallard, Velleman, Langsford, & Baldwin, 2001). Another investigation of children with injury showed that social withdrawal was related to concurrent PTSS, while resignation and social withdrawal were related to subsequent PTSS 6 months after injury (Marsac, Cirilli, Kassam-Adams, & Winston, 2011).

Two studies have examined appraisals and coping together with child PTSS in injured children. Stallard and Smith (2007) found that appraisals and coping (rumination, suppression, and distraction) together accounted for 64% of the variance in concurrent PTSS 8 months after injury (Stallard & Smith, 2007). Similarly, using structural equation modeling, Marsac et al. (2016) found that appraisals and coping (6–12 weeks post-injury) contributed to later child PTSS (6 months post-injury). Specifically, escape coping (a type of avoidant coping) mediated the relationship between threat appraisals and PTSS. Thus, while ample evidence supports a role for appraisals and coping in the development of PTSS, further research is needed to clarify the independent and interactional roles of these constructs, as well as the processes through which children form appraisals and coping strategies. We need such research to advance theoretical models and to inform intervention development for the prevention of PTSS in children exposed to PTEs.

Parents likely play a key role in helping their children recover following a PTE, but it has yet to be determined how this parental influence occurs. It has been well-established that parental psychological reactions (e.g., PTSS and depression) are modestly associated with child PTSS outcomes (Alisic et al., 2011; Morris, Gabert-Quillen, & Delahanty, 2012; Trickey et al., 2012). Gewirtz et al. (2008) suggested that parenting practices (not just parent symptoms/reactions to trauma) contribute to the child's functioning following a PTE. In the child anxiety literature, social learning models of child anxiety build on information-processing models by identifying patterns of parent–child interaction that promote (or challenge) children's maladaptive appraisals and avoidant coping strategies. These models have elucidated parent–child processes involved in maintaining child anxiety symptoms, often via interaction tasks that allow direct observation of moment-to-moment parent–child processes (Barrett, Rapee, Dadds, & Ryan, 1996; Chorpita, Albano, & Barlow, 1996; Cobham, Dadds, & Spence, 1999; Luis, Varela, & Moore, 2008). For example, in a study of 152 children and parents, parent–child processes maintaining anxiety were observed: parents of anxious children were more likely to reciprocate and reward avoidant coping suggestions made by their children, and when parents did this children were more likely to sustain avoidant coping strategies (Dadds, Barrett, Rapee, & Ryan, 1996). Parent coping assistance has been found to influence child coping with PTEs such as community violence exposure and natural disasters (Kliewer et al., 2006; Prinstein, La Greca, Vernberg, & Silverman, 1996).

To date, much research on appraisals and coping has relied on self-report and very little has examined the role of parental influence on children's appraisals and coping behaviors. To our knowledge, only two parent–child observational methods (neither focused on appraisals or coping) have been implemented with children exposed to PTEs. Gewirtz, DeGarmo, and Medhanie (2011) administered a family interaction task (including a fun activity, problem-solving activities, and a cooperation/competition activity) in the research lab, to assess mothers’ parenting practices following their child's exposure to intimate partner violence. Tasks were coded for positive involvement, problem-solving outcome, skill encouragement, and inept discipline. Results illustrated that mothers’ observed parenting predicted the child's trauma-related distress and fears (but not depression). Researchers are applying a novel methodology [i.e., Electronically Activated Recorder – EAR (Mehl, Pennebaker, Crow, Dabbs, & Price, 2001)] to assess child–parent communication in their natural environment, following a pediatric injury (Alisic, Barrett, Bowles, Babl, et al., 2015; Alisic, Barrett, Bowles, Conroy, & Mehl, 2015). Child–parent communication is recorded, for 30-sec every 5 min, for 2 days post-hospital discharge for pediatric injury. This new method examines parent–child communication in a natural setting, with particular attention to discussions around psychological recovery related to the injury event (Alisic, Barrett, Bowles, Babl, et al., 2015; Alisic, Barrett, Bowles, Conroy, & Mehl, 2015). Study results are forthcoming. The Gewirtz and Alisic studies provide examples of the unique, relevant information that can be obtained from direct observation of parent–child interactions.

With exposure to acute, single-incident trauma unfortunately common for children, a key goal is to identify etiological mechanisms which may be malleable in the early posttrauma period. Inspired by previous research conducted in the child anxiety literature (e.g., Barrett et al., 1996; Dadds et al., 1996) and with the goal of beginning to fill the gaps in the trauma field regarding observational assessment tools, our team created a parent–child interaction assessment tool (i.e., TAST) for children who have been exposed to trauma (see Method section for a description of the TAST). The purpose of this new observational assessment tool (i.e., the TAST) is to measure appraisals, coping solutions, and how parent–child interactions influence appraisals and coping solutions in families in which children have been exposed to acute trauma. The objectives of this manuscript are twofold: (1) to describe the newly created TAST and (2) to report on initial feasibility and validation of TAST implemented with child–parent dyads in which children were exposed to a PTE (i.e., children hospitalized for injury). Given that acute and chronic injury and illness and their associated medical care are among the most frequent PTEs experienced by children worldwide, we selected to first examine the TAST in a population of children with injuries and their parents (Murray & Lopez, 1996).

## Method

### Participants

As part of a larger study investigating biopsychosocial factors as predictors of PTSS following pediatric injury, children who were hospitalized for injury and their parents completed a three-module task (i.e., TAST). Participants were recruited while receiving treatment for an injury at a Level I Pediatric Trauma Center in the northeastern United States. Study inclusion criteria required that (1) youth were between 8 and 13 years of age, (2) had incurred an injury within the past 2 weeks which the child perceived as a PTE, (3) were currently hospitalized for treatment of their injury, (4) had a current Glasgow Coma Scale score >12, (5) had a parent who agreed to participate, and (6) had sufficient English language proficiency and cognitive ability to comprehend and answer questions. Youth were excluded from participating if injuries resulted from family violence or abuse. A total of 25 children (aged 8–13, *M*=10.4, SD=1.6) and one parent per child were enrolled in this study. All children and parents completed their participation during the child's inpatient hospitalization, within 2 weeks of injury (*M*=2.7, SD=2.8 days post-injury). Of 200 families deemed potentially eligible to participate in the study, over half (*n*=101) were missed by the research team prior to discharge, 2 did not perceive their injury event as potentially traumatic thus became ineligible for the full study, and 67 families elected not to participate in the study, resulting in a 13% enrollment rate. Primary reasons for the team missing eligible families included short hospitalizations, required medical procedures at the time of the research approach, and unavailability of parents for consent and participation. Reason for refusal included disinterest, child fatigue, child not feeling well, and child or parent not wanting to be video-recorded. See Table 1 for sample demographic characteristics.

**Table 1:** Demographics and event characteristics

Variable	*n*=25
Child age in years, *M* (SD)	10.4 (1.6)
Child sex—male, *N* (%)	19 (76.0)
Child race, *N* (%)	
Black/African American	11 (44.0)
White	11 (44.0)
Other	3 (12.0)
Child ethnicity, *N* (%)	
Hispanic	2 (8.0)
Non-Hispanic	23 (92.0)
Child injury type, *N* (%)	
Fracture	14 (56.0)
Concussion	3 (12.0)
Hemorrhage	2 (8.0)
Other	6 (24.0)
Method of injury, *N* (%)	
Recreational activity (e.g., playground, bike)	13 (52.0)
Sports (i.e., game or practice)	7 (28.0)
Motor-vehicle accident	4 (16.0)
Injured by animal	1 (4.0)
Participating parent relationship to child, *N* (%)	
Mother	21 (84.0)
Father	4 (16.0)
Participating parents age in years, *M* (SD)	41.1 (6.3)

### Procedure

Potential participants were identified using hospital records. Research assistants (RAs) approached caregivers of potentially eligible child participants in children's hospital rooms when children were not otherwise engaged in medical treatment. Caregivers first provided consent (and children provided assent) to an initial screening assessment to determine whether the child met the additional inclusion criterion of perceiving the injury event as potentially traumatic. Children then completed a validated four-item screen derived from the Acute Stress Checklist for Children (Kassam-Adams, 2006). For those children who screened positive, parental consent and child assent were obtained for the full study. Next, children and parents completed module 1 (interview assessment) of the TAST independently of each other. They then completed module 2 (parent–child discussion) together. Finally, the child completed module 3 (repeat brief version of interview assessment). Child and parent participants were offered US$15 each to thank them for their time. All research procedures were conducted in accordance with an institutional review board–approved study protocol.

### Measures

RAs collected demographic information from parents and children and abstracted information related to the injury event from the medical record.

#### Trauma ambiguous situations tool

We created the TAST for children exposed to a PTE by adapting ambiguous situation task methodology previously used by several researchers to assess parent–child processes related to appraisals and coping in child anxiety or oppositional behaviors (Barrett et al., 1996; Chorpita et al., 1996; Dadds et al., 1996; Luis et al., 2008; Varela et al., 2004; Varela, Niditch, Hensley-Maloney, Moore, & Creveling, 2013). The original procedures were created by Barrett et al. (1996) in a set of studies in which they presented a series of hypothetical scenarios separately to children and parents to elicit cognitive appraisals (threat versus neutral interpretations of an ambiguous situation) and coping solutions (adaptive versus maladaptive). Procedures included 12 ambiguous situations with a focus on potential social or physical threat. In Barrett et al.'s task, for each scenario, children and parents provided one free response appraisal, selected from one forced-choice appraisal, and provided one free choice coping solution. Then, children and both parents engaged in four family discussions: one social threat, one physical threat, one child-directed “hot topic,” and one parent-directed “hot-topic.” Following the family discussion, children provided a final free choice coping solution. In addition, the family discussion task was coded for process variables to enable the examination of the interaction between parents and children (Barrett, 1996; Barrett et al., 1996; Dadds et al., 1996; Dadds, Ryan, Barrett, & Rapee, 1992). Other child anxiety investigators have varied the number of scenarios (1–12) and response options (e.g., list as many appraisals and coping solutions as possible and pick the most likely; Chorpita et al., 1996; Luis et al., 2008; Varela et al., 2004; Varela et al., 2013).

The newly adapted TAST includes three modules: two interview assessments and a parent–child discussion task. See Table 2 for an example of a TAST scenario and response choices. Following procedures originated by Barrett (1996), in module 1 (interview assessment), the child and parent are separately presented with ambiguous situations (Barrett, 1996; Barrett et al., 1996). To reduce participant burden, we limited the interview to four ambiguous situations: The first situation (“When you wake up tomorrow morning, you notice your tummy feels funny”) was modified from one of the hypothetical situations created by Barrett (1996) to anchor the TAST in the child anxiety literature (Barrett, 1996). We created three new ambiguous situations that could be related to the child's PTE (i.e., injury and medical treatment) or his/her reaction to it. For example, “You are alone in your room and notice that your heart is beating fast.” TAST response options for each ambiguous situation begins with open-ended responses from the child or parent (appraisals and coping solutions), followed by the respondent's selection of the “most likely” appraisal and coping solution from those he or she generated, and finally, selection from a forced-choice appraisal question (two threat and two neutral, in random order; Barrett, 1996; Barrett et al., 1996; Chorpita et al., 1996; Varela et al., 2004; Varela et al., 2013).

**Table 2:** An example of a scenario with response choices from Trauma Ambiguous Situations Tool (TAST)*

Scenario: When you wake up tomorrow morning, you notice your tummy feels funny.	
Module 1: Interview Assessment**	
Child	Domain 1: Appraisals
	Open ended: What do you think could be happening? Which of these reasons/explanations do you think is most likely?
	Forced choice:
	1. You might be hungry (neutral).
	2. You ate some bad food and you are going to be really sick (negative trauma cognition).
	3. It's okay. It will go away soon (neutral).
	4. There is something wrong with your stomach and you will need a big operation (negative trauma cognition/current—unrealistic—threat).
	Domain 2: Coping Behavior
	If you woke up and noticed your tummy feels funny, what are some things you could do? What would you most likely do if this happened?
Module 2: Parent–child Discussion Task	
Child/Parent	Discuss this situation with each other. Discuss what could be happening. Discuss some things you [CHILD] can do.
Module 3: interview assessment	
Child	Domain 1: Appraisals
	Open ended: Why does your tummy feel funny? Please give me a final answer of what is most likely happening.
	Forced choice:
	1. There is something wrong with your stomach and you will need a big operation (negative trauma cognition/current—unrealistic—threat).
	2. It's okay. It will go away soon (neutral).
	3. You ate some bad food and you are going to be really sick (negative trauma cognition).
	4. You might be hungry (neutral).
	Domain 2: Coping Behavior
	What would you do if you woke up and noticed your tummy feels funny? Please give me a final answer of what you would most likely do if this happened.

* Detailed instructions for task administration (including prompts) and the code book are available from authors

** parent module 1 instructions are parallel to the child's instructions.

In module 2 of the TAST (parent–child discussion task), the child and parent are brought together for a discussion of two of the scenarios. Following Barrett et al. (1996) and Chorpita et al. (1996), we instructed children and parents to discuss both what was happening in the situations (i.e., their appraisal) as well as how to deal with the situation (coping solution).

In module 3 (interview assessment), immediately following each parent–child discussion, we asked the child to provide a single final appraisal (first open-ended, then forced choice) and single final coping solution (open-ended) for this scenario. In our study, all modules were audio- and video-recorded and transcribed.

Two trained RAs worked together to administer the TAST. First, for module 1, child and parent participants were separated and interviewed independently following the flow described in the example in Table 2: First, participants were presented with a scenario (e.g., When you wake up tomorrow morning, you notice your tummy feels funny). Next, they were asked to generate open-ended appraisals and then select from a list of possible appraisals. Then, they were asked to generate open-ended coping solutions. In module 2, the child and parent were brought back together, presented with a scenario, and asked to discuss appraisals and coping solutions together. Finally, in module 3, the child was asked to generate a final appraisal (open-ended), select a final appraisal from forced-choice options, and provide a final coping solution (open-ended).

We coded child and parent utterances using the Family Anxiety Coding Schedule, which provides codes for each utterance to denote (1) the speaker and to whom it is directed, (2) process (facilitate, hinder, listen, respond, reassurance, and question), (3) content (neutral or threat problem description; proactive or avoidance problem solution; positive or negative consequence of problem solution), and (4) affect (happy, anxious, sad, angry, and neutral; Barrett, 1996; Dadds et al., 1992). Each child and parent utterance was coded for content, process, and affect, as relevant. Each utterance was coded with all relevant codes (i.e., an utterance could have more than one code). Two trained RAs coded every interview and discussion independently. Coders then met together to review assigned codes, and any discrepancies were resolved by team consensus (Armstrong, Gosling, Weiman, & Martaeu, 1997). Detailed TAST administration instructions and the codebook are available from authors.

## Results

### Feasibility of the TAST

Once families agreed to participate in the study, almost all completed the TAST (two families were withdrawn from the study: one was discharged following the module 1 interview and elected to leave before finishing the TAST, one enrollment was interrupted by the medical team and child fatigue). Audio-recording was successful in all but one interview assessment. Video-recording was successful in 23 (92%) of the cases; two recordings were lost due to equipment failure. Initial interview assessments averaged approximately 12 min for children and 13 min for parents. Parent–child discussions lasted an average of 2 min for each scenario, though dyads were prompted to continue when discussions were less than 5 min. Child module 3 interview assessments were very quick, generally lasting 1 min or less. Children and parents needed very few prompts (other than letting them know that they had more time) during the discussion task (scenario 1: *M*=2.3, SD=2.4, mode=2, range 0–12; scenario 2: *M*=1.7, SD =1.2, mode=2, range 0–5). Types of prompts included reminding children and parents that they had more time to continue the discussion (most frequent prompt) and clarifying instructions. In general, children and parents expanded their discussion about 25% of the time when told that they had more time to continue the parent–child discussions.

### Initial development and validation of the TAST

#### Engagement in TAST

Children and parents appeared to be genuinely engaged in the discussion task as evidenced by staff observation and the number of utterances that they each made related to appraisals and coping as presented in Table 3. See Table 4 for examples of the application of appraisal and coping codes. In addition, parents and children were engaged with each other in helping the child come to a final appraisal and final coping solution. An example parent–child interaction discussing the child's tummy feeling funny is as follows:

Parent: What could be happening if your tummy feels funny? What do you think?

Child: Hungry.

Parent: Oh okay! Is that the main thing you would think, is that you felt hungry?

Child: I ate a lot of junk food.

Parent: Really?! Oh my gosh! See, you know what I would think? I was thinking that maybe were having a reaction from your anesthesia, and the concussion or something with the surgery was the reason why. And then I thought that maybe it could be that you have to go to the bathroom and – But I never thought for a second that you were hungry or that you ate something funny.

Child: Yeah.

Parent: I thought the exact opposite. So what do you think now? Do you think keep your idea or do you think it could have something to do with your surgery?

Child: Maybe the surgery.

Parent: I mean, I know because I'm your mom, because that's how I see it, I just think right now that anything that you do or say that feels funny, I'm going to connect it with something that happened with your surgery. But I think that's kind of interesting because you think the opposite, it had nothing to do with it.

**Table 3:** The number of child and parent utterances related to appraisals and coping during Module 2 (parent–child discussion) portion of the TAST

	Scenario 1 *M* (SD), mode, range	Scenario 2 *M* (SD), mode, range
Child utterances: appraisals	3.2 (2.0), 3, 0–9	3.8 (2.3), 4, 0–9
Child utterances: coping	5.4 (4.9), 3, 0–21	2.8 (1.4), 3, 1–6
Parent utterances: introduce appraisals	2.6 (2.7), 1, 0–9	2.0 (2.0), 1, 0–9
Parent utterances: reinforce child appraisals	1.1 (1.4), 0, 0–6	1.4 (2.0), 0, 0–7
Parent utterances: introduce coping strategies	3.7 (3.3), 1, 0–11	1.6 (1.4), 0, 0–4
Parent utterances: reinforce to coping strategies	1.9 (2.6), 0, 0–9	0.88 (1.0), 0, 0–4

**Table 4:** Sample utterances with appraisal and coping codes

Child examples			
Child age	Child sex	Quote	Codes
11	M	I'm playing and, and my heart's beating fast because of how hard I played.	Neutral appraisal
10	F	The medicine is having some effect on me.	Threat appraisal
8	F	I would say, um why do you need to talk to my mom and dad? Is there something wrong with me?	Proactive solution
10	M	Try not to listen because I don't want to hear what he has to say.	Avoidant solution

Parent examples			

Participating parent		Quote	Codes
Mother		She may have to go to the bathroom.	Neutral appraisal
Mother		Maybe he's having an anxiety attack.	Threat appraisal
Father		Hit the button for the nurse to come.	Proactive solution
Father		Try to stop thinking about what's wrong with his arm.	Avoidant solution

### Variation and change in appraisals and coping solutions

Results of interview assessments and the discussion task demonstrated a reasonable amount of variation in child and parent responses. Tables 5 and 6 represent the number of unique appraisals and coping solutions offered by children and parents during each TAST module. These data demonstrate the value in collecting both open-ended and forced-choice appraisals from children and parents, as differences in final answer appraisal choices were observed based on forced choice versus open choice. More children (in three out of four scenarios) and parents (in all scenarios) offered threat appraisals as final choices in their open-ended responses compared to their selection among forced-choice responses (See Tables 5 and 6).

**Table 5:** Unique child appraisals and coping solutions in each TAST module

		Q1: Tummy funny	Q2: Machine in room	Q3: Heart beating fast	Q4: Doctor talk to parents
Module 1: Interview Assessment	Appraisals				
	Open-ended				
	Neutral, *M* (SD), range	1.0 (1.0), 0–3	1.4 (1.1), 0–4	0.6 (0.6), 0–2	1.3 (1.1), 0–4
	Threat, *M* (SD), range	1.3 (0.9), 0–4	0.7 (0.6), 0–2	1.4 (0.8), 0–3	1.1 (0.7), 0–3
	Neutral:threat ratio	10:13	2:1	3:7	13:11
	Final answer threat, *N* (%)	14 (60.9)	6 (26.1)	13 (61.9)	10 (45.5)
	Forced choice				
	Threat, *N* (%)	4 (16.0)	7 (28.0)	6 (24.0)	3 (12.0)
	Coping Solutions				
	Proactive, *M* (SD), range	2.4 (1.0), 1–4	1.4 (1.0), 0–4	2.3 (1.3),1–5	1.3 (0.8), 0–3
	Avoidant, *M* (SD), range	0.2 (0.5), 0–2	0.2 (0.5), 0–2	0.1 (0.3), 0–1	0.3 (0.7), 0–3
	Proactive:avoidant ratio	12:1	7:1	23:1	13:3
	Final answer avoidant, *N* (%)	1 (4.2)	1 (5.3)	1 (4.3)	5 (22.7)
Module 2: Parent–Child discussion	Appraisals (utterances)				
	Neutral, *M* (SD), range	0.92 (0.81), 0–2	N/A	N/A	1.3 (1.3), 0–5
	Threat, *M* (SD), range	1.0 (0.76), 0–3	N/A	N/A	1.2 (0.96), 0–4
	Neutral:threat ratio	23:25	N/A	N/A	13:12
	Coping Solutions (utterances)				
	Proactive, *M* (SD), range	2.4 (1.8), 0–6	N/A	N/A	1.2 (1.1), 0–4
	Avoidant, *M* (SD), range	0.12 (0.33), 0–1	N/A	N/A	0.20 (0.50), 0–2
	Proactive:avoidant ratio	20:1	N/A	N/A	10:1
Module 3: Interview Assessment	Appraisals				
	Open-ended				
	Final answer threat, *N* (%)	13 (52.0)	N/A	N/A	8 (33.3)
	Forced-choice threat, *N* (%)	5 (20.8)	N/A	N/A	3 (12.0)
	Coping solutions				
	Final answer avoidant, *N* (%)	0 (0.0)	N/A	N/A	3 (12.0)

**Table 6:** Unique parent appraisals and coping in TAST modules 1 and 2

		Q1: Tummy funny	Q2: Machine in room	Q3: Heart beating fast	Q4: Doctor talk to parents
Module 1: Interview	Appraisals				
Assessment	Open-ended				
	Neutral, *M* (SD), range	0.88 (0.83), 0–3	2.0 (1.2), 0–6	0.60 (0.76), 0–2	1.9 (1.2), 0–4
	Threat, *M* (SD), range	1.7 (1.1), 0–5	0.83 (1.1), 0–5	1.8 (1.1), 0–4	0.80 (1.3), 0–6
	Neutral:threat ratio	22:43	200:83	1:3	12:5
	Final answer threat, *N* (%)	19 (79.2)	2 (9.5)	18 (81.8)	6 (25.0)
	Forced choice				
	Threat, *N* (%)	3 (12.0)	1 (4.0)	5 (20.0)	4 (16.0)
	Coping solutions				
	Proactive, *M* (SD), range	3.4 (1.2), 1–6	1.9 (0.93), 1–4	2.4 (1.2), 1–6	1.7 (0.98), 0–3
	Avoidant, *M* (SD), range	0.16 (0.37), 0–1	0.20 (0.65), 0–3	0.08 (0.28), 0–1	0.56 (1.1), 0–4
	Proactive:avoidant ratio	85:4	19:2	30:1	17:6
	Final answer				
	Avoidant, *N* (%)	0 (0.0)	1 (4.0)	0 (0)	4 (17.7)
Module 2: Parent–Child discussion	Appraisals (utterances)				
	Introduce neutral appraisals				
	*M* (SD), range	0.84 (1.2), 0–5	N/A	N/A	1.2 (1.1), 0–4
	Introduce threat appraisals				
	*M* (SD), range	0.96 (1.0), 0–4	N/A	N/A	0.56 (0.96), 0–4
	Neutral:threat ratio	7:8	N/A	N/A	15:7
	Reinforce neutral appraisals				
	*M* (SD), range	0.48 (0.77), 0–3	N/A	N/A	0.96 (1.7), 0–7
	Reinforce threat appraisals				
	*M* (SD), range	0.60 (0.96), 0–3	N/A	N/A	0.48 (0.71), 0–2
	Neutral:threat ratio	4:5	N/A	N/A	2:1
	Coping solutions (utterances)				
	Introduce proactive				
	*M* (SD), range	2.1 (1.4), 0–4	N/A	N/A	0.80 (0.71), 0–2
	Introduce avoidant				
	*M* (SD), range	0.08 (0.40), 0–2	N/A	N/A	0.36 (0.81), 0–3
	Proactive: avoidant ratio	105:4	N/A	N/A	20:9
	Reinforce proactive				
	*M* (SD), range	1.9 (2.6), 0–9	N/A	N/A	0.72 (0.98), 0–4
	Reinforce avoidant				
	*M* (SD), range	0.04 (0.20), 0–1	N/A	N/A	0.16 (0.47), 0–2
	Proactive:avoidant ratio	95:2	N/A	N/A	9:2

In addition to variation of responses, following module 2 (parent–child discussion) a number of children changed their answers in the module 3 assessment (compared to the module 1 assessment). More specifically, 11 (47.8%) children changed their answers for the final open-ended appraisals for question 1 (seven threat to neutral, four neutral to threat) and 8 (38.1%) children changed their answers for the final open-ended appraisal for question 2 (six threat to neutral, two neutral to threat). For forced-choice appraisal data, following the parent–child discussion, three (12.5%) children changed their responses on question 1 (one threat to neutral and two neutral to threat) and four (16%) children changed their responses on question 4 (two threat to neutral and two neutral to threat). Only a few children changed their answers for the final open-ended coping solutions (question 1: one avoidant to proactive; question 2: two avoidant to proactive).

## Discussion

Our study suggests that the TAST may be a valuable tool in advancing our understanding of how appraisals and coping solutions may contribute to the development and maintenance of PTSS, as well as how parents may shape children's appraisals and coping in the early aftermath of trauma. To our knowledge, this is the first study to apply this type of ambiguous situations methodology to assess appraisals and coping solutions and how these are influenced by parents in children exposed to a PTE. Implementation of the full TAST (two interview assessments, parent–child discussion task) was feasible, even in a peri-trauma context. In the particularly challenging context of a child's hospital room within a few days of injury, we did experience some enrollment obstacles. We found that once consent was obtained, TAST administration was straightforward and even enjoyable for many families.

The TAST provided novel information on child and parent appraisals and coping solutions that has not been available through standard self-report measures. In addition, the opportunity to observe how parents shaped their child's appraisals and coping solutions (e.g., offering appraisals, reinforcing children's appraisals) provided valuable information that, with additional research, may be infused into development of future interventions.

The current study extends the application of observational/ambiguous situations methodology such as that used in the TAST in two primary ways: (1) application to children exposed to a PTE rather than presenting with anxiety or oppositional behaviors and (2), application in a real-world setting (a child's hospital room) rather than a lab-based setting (Barrett et al., 1996; Chorpita et al., 1996; Dadds et al., 1996; Luis et al., 2008; Varela et al., 2004; Varela et al., 2013). The administration of the TAST was seamless for families who consented to study participation, with almost every family completing all modules. Child–parent dyads completed module 2 very quickly, typically taking only 2–3 min for each scenario, and children completed module 3 in less than a minute for each scenario. In our design of the TAST, we chose to limit modules 2 and 3 to just two scenarios to reduce participant burden; however, given the ease of administration, it is unlikely that administering all four scenarios would be problematic for families and the additional information garnered from including all scenarios may be valuable. Thus, in the future, researchers may want to include all of the scenarios that they use in module 1 also in modules 2 and 3 (recognizing the additional time needed for coding the open-ended responses).

By combining methodology used by several past researchers (Barrett et al., 1996; Chorpita et al., 1996; Dadds et al., 1996; Luis et al., 2008; Varela et al., 2004; Varela et al., 2013), we were able to identify differences in children's and parents’ responses (i.e., appraisals) with implications for psychological assessments more broadly. It may be that children and parents are more likely to provide a socially desirable response (i.e., non-threat response) when they are given specific options from which to choose. Alternatively, our forced-choice response options may not have included enough variation in types of appraisals of threat to capture children's or parents’ own thoughts and appraisals. Given the difference in responses and the wealth of information gathered from the open-ended responses, assessing appraisals with both of these two modalities may be of benefit. The open-ended responses provided more detailed information of what types of appraisals children and parents may naturally present, which may help to further inform future interventions while the forced-choice options allows for easier comparisons across groups of children. Administering the open-ended and forced-choice appraisals worked well and did not add substantial time to the research protocol. However, the coding of the open-ended appraisals was time intensive and ought to be a consideration in designing future studies. In addition, future research should examine whether one of these types of assessment is a “better” representation of appraisals. For example, do open-ended appraisals or forced-choice appraisals relate differentially to validated measures of global appraisals? Does either assessment of appraisals predict concurrent or subsequent PTSS?

Supporting Marsac et al.'s (2014) theory regarding parents’ role in the development of appraisals, results from the current study suggest that the conversation between children and parents during module 2 appears to influence children's appraisals. Approximately, 40–50% of children changed their open-ended response and 12–16% changed their forced-choice response of how they appraised the ambiguous situations. Children's appraisals changed in both directions (i.e., from neutral to threat and threat to neutral). Given the small sample size, we were unable to examine parental factors that may have influenced these changes (e.g., Did children change their responses to align with their parents? Did children change their responses based on what their parents reinforced or suggested during the discussion?). Future research, with larger samples, can help to clarify these questions. In addition, future research with the TAST may allow us to examine bi-directional processes between the parent and the child to better understand what parents do that influences change in their children as well as how children influence their parents during peri-trauma or around potential trauma triggers. Another avenue for future research with the TAST is to combine it with self-report and parent-report assessments of parent–child relationships. One measure of particular relevance is the Childhood Attachment and Relational Trauma Screen (CARTS), which can be used to assess the relational-socioecological context surrounding the child's trauma exposure (Frewen, Brown, DePierro, D'Andrea, & Schore, 2015). While developed specifically for child maltreatment, the measure may be able to be used with acute trauma types as well. Combining information provided by the TAST and the CARTS would offer a more comprehensive multi-method multi-informant assessment of parent–child interaction posttrauma. Better understanding of parent–child interaction posttrauma exposure has implications for intervention development and clinical treatment: if we know how parents influence their child's appraisals following trauma exposure, we can better determine how to intervene.

Interestingly, both children and parents predominantly offered proactive coping solutions, both proportionally (compared to avoidant solution) and as final answers. While we do not know how this choice relates to PTSS in the current sample, past work suggests that avoidant coping relates to PTSS (Marsac et al., 2016). In addition, past research has suggested that parents’ own avoidance following trauma exposure may influence their interactions with their family. For example, in a recent observational study of post-deployment family interactions, Brockman et al. (2016) found that experiential avoidance (and PTSS) in male military members was related to less positive engagement with spouses and children. Experiential avoidance was also related to more avoidance of distress (i.e., children/spouses exhibiting aversive behaviors or affective distress). So, while only a few child–parent dyads committed to avoidant coping solutions in this study, this may be an indicator of risk for future PTSS in children and/or parents and/or have implications for family functioning. Parents who experience avoidance themselves may be more likely to transmit this approach of processing traumatic events to their children. Future research will help us determine if the TAST can also help identify children at-risk for PTSS based on coping choices. Parents influence on coping solution is less clear in the current sample as most parents and children selected proactive solutions from the onset. A larger sample size may help to determine if there are specific processes or factors associated with parents either encouraging a proactive or avoidant coping solutions.

While the current study findings suggest that the TAST may be a promising tool to help better understanding children's appraisals and coping following a PTE, several limitations should be noted. First, the data we present here are from a small sample of children, all whom experienced injury as a PTE, and most parent participants were mothers. Future research should examine the applicability of the TAST with a larger sample of children and with additional trauma types to expand the generalizability of results. In addition, inclusion of more fathers in future samples would allow for comparisons of how mothers and fathers may socialize appraisals and coping posttrauma exposure in their children differently. Currently, the conclusions that we present with the TAST are limited primarily to its use with mothers, given the small number of fathers who participated in this study. Although we piloted the TAST with children exposed to injury, the TAST can readily be adapted for a number of types of PTEs by using the methodology outlined in this paper and modifying the ambiguous situations. Second, the enrollment rate of 13% is lower than desired and could suggest a selection bias in those who chose to participate in the study. However, this rate is only slightly lower than other studies of children hospitalized for injury or acute illness (e.g., 25% and 34%), suggesting that the enrollment challenges were likely due in part to recruiting in pediatric hospital setting rather than to the nature of the TAST itself (Kassam-Adams et al., 2011, 2015). To try to improve enrollment rates in the future, given the experience of families who completed the TAST, researchers may want to emphasize that families often find that participating in the TAST is simple, quick, and sometimes enjoyable. In addition, it would be helpful for future research to characterize families that agree to participate to better inform generalizability. Finally, this was an initial study with the TAST, and we do not yet know how the appraisals and coping constructs assessed by the TAST relate to other measures of appraisals, coping, or concurrent or subsequent PTSS. Thus, examining how the appraisals and coping assessed by the TAST and how child–parent process is related to PTSS is an important avenue for future work.
